# Cochlear signal intensity changes in vestibular schwannoma: a balanced fast field-echo MRI study

**DOI:** 10.3389/fneur.2025.1549869

**Published:** 2025-05-19

**Authors:** Hiroko Takeda, Takeshi Fujita, Tomonori Kanda, Yoichi Uozumi, Natsumi Uehara, Jun Yokoi, Akinobu Kakigi, Takashi Sasayama, Ken-ichi Nibu

**Affiliations:** ^1^Department of Otolaryngology-Head and Neck Surgery, Kobe University Hospital, Kobe, Japan; ^2^Department of Radiology, Kobe University Hospital, Kobe, Japan; ^3^Department of Neurosurgery, Kobe University Hospital, Kobe, Japan

**Keywords:** vestibular schwannoma, sensorineural hearing loss, MRI, bFFE, cochlea

## Abstract

**Introduction:**

Vestibular schwannoma (VS) is a benign tumor associated with cochlear degeneration and sensorineural hearing loss (SNHL). This study aimed to evaluate cochlear signal intensity in VS patients using balanced fast field-echo (bFFE) magnetic resonance imaging (MRI) and to explore its relationship with tumor size, hearing loss, and postoperative changes.

**Methods:**

A retrospective analysis was conducted on 165 VS patients and 30 SNHL control patients who underwent bFFE MRI at Kobe University Hospital from 2008 to 2019. Cochlear signal intensity was measured bilaterally using regions of interest (ROIs), and signal intensity ratios (affected/normal) were calculated. Statistical analyses included linear regression and ANOVA to evaluate correlations with hearing levels, tumor size, and postoperative changes.

**Results:**

VS patients exhibited significantly lower cochlear signal intensity ratios on the affected side compared to controls (75.3% vs. 100%, *p* < 0.0001). Correlations between cochlear signal intensity ratios and hearing levels were significant in Koos grade I tumors but not in higher-grade tumors. Tumor size was negatively correlated with cochlear signal intensity ratios in Koos grade II–IV tumors. Postoperatively, signal intensity normalized within 1–2 years, irrespective of hearing preservation.

**Discussion:**

Decreased cochlear signal intensity in VS patients may indicate protein concentration changes due to tumor secretions. bFFE MRI effectively captures these changes, providing insights into cochlear physiology and tumor impact.

**Conclusion:**

bFFE MRI is a reliable tool for assessing cochlear signal intensity in VS patients, offering potential for improved tumor evaluation, surgical planning, and postoperative monitoring.

## 1 Introduction

Vestibular schwannoma (VS) is a benign tumor that develops from the Schwann cell sheath surrounding the vestibular nerve. It typically arises from the glial-Schwann sheath junction, known as the Obersteiner–Redlich zone, and is usually found at or near the porus of the internal auditory canal (1). VS is known to cause progressive sensorineural hearing loss (SNHL). There is significant degeneration of cochlear tissue in ears with VS, which is believed to contribute to hearing loss (2). However, the cochlear degeneration cannot be solely attributed to pressure from the tumor. Several hypotheses have been proposed to explain the causative factors, including the secretion of ototoxic molecules by VS (3), neuroinflammation, fibrosis, and edema within the tumor microenvironment (4). Nevertheless, the exact cause of the degeneration remains unknown (5). Additionally, about 20% of VS patients experience sudden hearing loss without apparent changes in tumor configuration (6). This poses challenges in managing hearing loss in VS patients.

Numerous studies have utilized magnetic resonance imaging (MRI) scans, particularly fluid-attenuated inversion recovery (FLAIR) sequences, to examine the cochlea in VS patients (7–11). Notably, it has been reported that an elevated FLAIR signal in the affected cochlea demonstrates a sensitivity of 80% and specificity of 95% (7). This change in cochlear signal intensity may represent an alteration in cochlear protein concentration caused by VS secretions. However, while higher preoperative FLAIR signals tend to correlate with poorer hearing outcomes, this correlation has not yet been shown to be statistically significant. Therefore, there is growing interest in the possibility that MRI signals in the cochlea reflect changes, such as protein concentration, within the cochlea (8).

In recent years, balanced fast field-echo (bFFE) sequences have gained popularity for evaluating the internal auditory canal and cochlea. bFFE sequences can quickly capture images while suppressing flow artifacts, and the signal intensity is proportional to T2/T1 (12). Notably, structures other than water exhibit low signal intensity, enabling the bFFE sequence to visualize subtle compositional changes in the cochlea without the need for contrast media. Previous FLAIR studies have acquired images with a slice thickness of 5 mm, which is often insufficient for evaluating the small structures of the cochlea. In contrast, the bFFE sequence acquires images at a thinner slice thickness of 1.4 mm in a short acquisition time. This advantage allows for a more accurate evaluation of subtle cochlear changes. Furthermore, the high contrast and rapid imaging capability of bFFE enhance its utility in detecting minute alterations in the cochlear microenvironment that may be overlooked with FLAIR. However, only a few studies have investigated the VS cochlea using bFFE sequences. Therefore, this study aims to evaluate the cochlear signal intensity in VS patients using bFFE sequences and explore the underlying causes of hearing loss.

## 2 Materials and methods

### 2.1 Patients

A retrospective review was conducted on 198 patients with cerebellopontine angle (CPA) tumors who underwent CPA MRI at Kobe University Hospital between 2008 and 2019. These patients were classified as the “VS group.” Patient charts and MRI images were examined to exclude those with no mention of suspected VS or other diagnoses such as meningioma. Patients without bFFE sequence images, bilateral VS, and those diagnosed with neurofibromatosis type 2 were also excluded. Among the VS group, patients who underwent a hearing test were designated as the “VS group with PTA.” For the control group, 30 patients with unilateral SNHL who did not have VS were included. They underwent CPA MRI using the bFFE sequence at Kobe University Hospital between 2008 and 2019. To be considered as having unilateral SNHL, patients needed to have a difference of 10 decibels hearing level (dB HL) or more between their left and right ears. Patients with SNHL attributable to identifiable MRI findings, such as tumors in the cerebellopontine angle, were excluded from the SNHL group. This study was approved by Kobe University Graduate School of Medicine Institutional Review Board (#B230044).

### 2.2 Radiologic analysis

**Figure 1:**
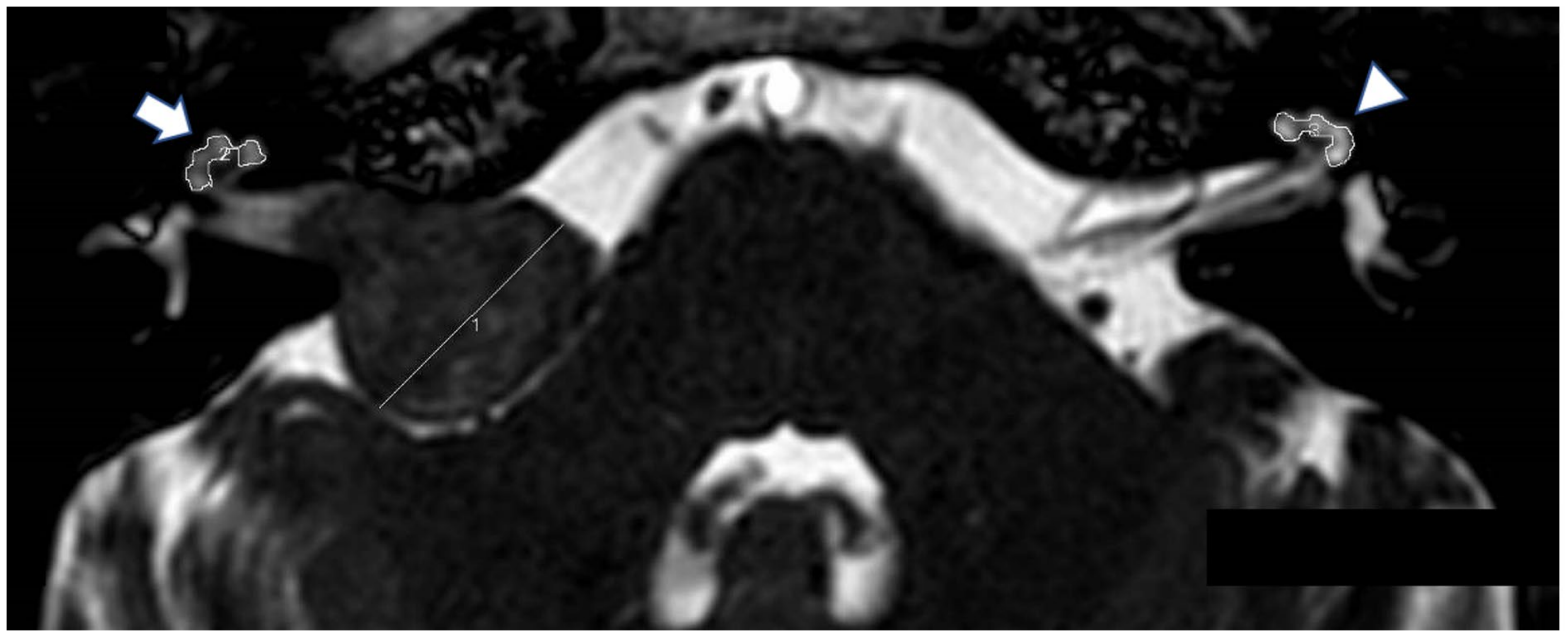
Balanced fast field-echo (bFFE) sequence showing regions of interest (ROIs) and the tumor diameter. The MRI image displays hand-drawn ROIs around the affected cochlea (arrow) and the normal cochlea (arrowhead).

3D bFFE was performed on a 3 T MR unit (Achieva; Philips Medical Systems) with the following parameters: TR/TE = 5.56/2.28 ms, flip angle = 45°, slice thickness = 1.4 mm, field of view = 150 × 150 mm, matrix = 512 × 512, and acquisition time = 1 min. The data extraction process was conducted by H.T., who was blinded to the patients’ clinical details, including hearing test results. We evaluated the mean signal intensities of the basal and middle cochlear turns bilaterally using a region-of-interest (ROI) (Figure 1). The cochlear signal intensity ratio was calculated by dividing the MRI signal intensity of the affected cochlea by the signal intensity of the normal cochlea. This ratio was used to compare the signal intensity between the affected and normal sides in the study population. The signal intensity was measured on preoperative MRI. In cases where surgery was performed, both the preoperative and postoperative signal intensities were measured. We also evaluated the mean signal intensity in the cochlea for the SNHL group. Additionally, we measured the maximum diameter of the tumor using the Koos grading scale (grade 1: small intracanalicular tumor, grade 2: small tumor with protrusion into the CPA without contact with the brainstem, grade 3: tumor occupying the cerebellopontine cistern without displacement of the brainstem, grade 4: large tumor with displacement of the brainstem and cranial nerves) (13). Multiple MRI slices displaying the tumor were reviewed, and the largest dimension observed was recorded as the maximum diameter. All measurements were performed using Fujifilm Shade Quest ViewR (Fujifilm Corporation, Tokyo, Japan).

### 2.3 Hearing outcomes

Audiometry was performed by experienced audiologists using a pure-tone audiometer (AA-H1 RION Co., Japan) in a soundproof booth. Pure-tone thresholds were obtained at 500, 1,000, and 2,000 Hz to calculate three-tone pure-tone averages (PTA). For patients with a PTA of “no response,” a hearing level of 110 decibels (dB HL) was recorded. Audiograms were obtained from the most recent date of MRI imaging. Patients who maintained a three-tone PTA <40 dB HL on the postoperative hearing test were considered to have hearing preservation.

### 2.4 Statistical analysis

The cochlear signal intensity ratio in the VS group was compared to the reference ratio (normal side), which had a constant value of 100%, using a one-sample *t*-test. To compare the cochlear signal intensity among the Koos I, Koos II–IV groups and SNHL groups, one-way ANOVA test was performed. The correlation between the cochlear signal intensity ratio on the affected side of VS patients and their average hearing level measured by PTA, as well as the correlation between tumor size and cochlear signal intensity ratio, were analyzed using linear regression analysis. The coefficient of determination (R^2^) was calculated. The regression equation used was y = mx + b, where y is the cochlear intensity ratio and x is the average hearing level or tumor size, as appropriate. Cochlear signal intensity ratios were compared among the pre-surgery group and different post-surgery time points using one-way ANOVA followed by Dunnett’s multiple comparisons test. All statistical analyses were performed using GraphPad Prism 10 (GraphPad Software, San Diego, CA, United States). All tests were conducted with a significance level of *p* < 0.05.

## 3 Results

### 3.1 Demographics

Detailed patient demographics are shown in Table 1. Of the 198 patients with CPA tumors, 30 patients with meningiomas and 11 patients with bilateral VS were excluded. In total, 165 VS patients met the inclusion criteria, of which 69 were male and 96 were female. The mean age was 51.7 years, ranging from 15 to 90 years. Of these, 118 out of 165 (71.5%) had their hearing tested. The mean PTA on the affected side and normal side was 54.1 ± 33.7 (mean ± SD) dB HL and 17.2 ± 12.6 dB HL, respectively. The tumor size was classified as Koos grade 1 in 24 cases and Koos grade 2–4 in 114 cases. Among the 165 patients, 111 (67.3%) underwent surgery, all using a suboccipital approach. Among them, there were only 5 cases of VS with Koos grade 1.

**Table 1:** Demographic and clinical characteristics of the patients with vestibular schwannoma (VS) or unilateral sensorineural hearing loss (SNHL).

	VS (*n* = 165)	Unilateral SNHL (*n* = 30)
Age (years, mean ± SD)	51.7 ± 15.7	60.1 ± 19.3
Sex (Male/Female)	69/96	11/19
Affected side (Right/Left)	80/85	16/14
Tumor size (Koos grade 1/2–4)	24/141	n/a

There were 30 unilateral SNHL patients identified who underwent CPA MRI (using the bFFE sequence). Of the 30 included patients, 11 were male and 19 were female. The mean age was 60.1 years, ranging from 11 to 85 years. The causes of SNHL were Meniere’s disease (*n* = 8), vertigo (*n* = 8), labyrinthitis (*n* = 1), and unknown etiology (*n* = 13). The mean PTA on the affected side and normal side was 57.1 ± 26.1 dB HL and 22.5 ± 14.6 dB HL, respectively.

### 3.2 Mean signal intensity in the cochlea

**Figure 2:**
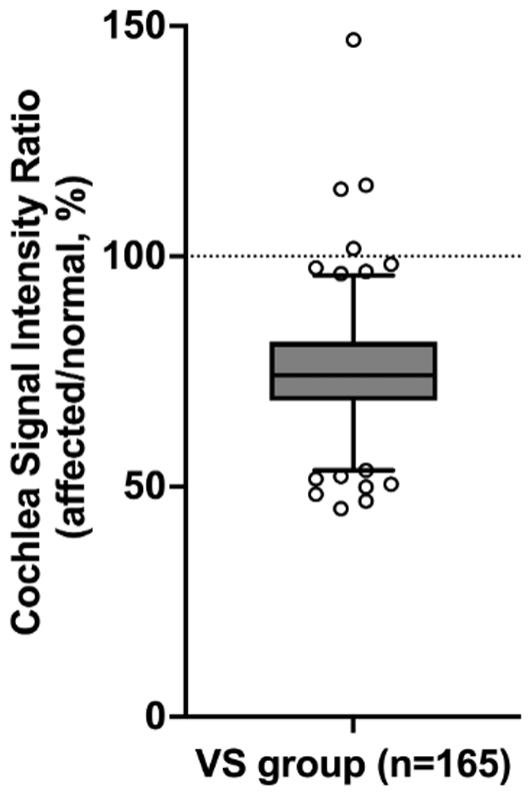
Comparison of the cochlear signal intensity ratio on the affected and normal sides. The cochlear signal intensity ratio represents the percentage of the MRI signal intensity of the cochlea on the side with vestibular schwannoma (VS) (affected side) relative to the signal intensity of the cochlea on the normal side (normal side). In 161 out of 165 VS patients, the mean signal intensity in the cochlea was lower on the affected side than on the normal side. The VS group shows a significantly lower cochlear signal intensity ratio (75.3%) compared to the reference value (*p* < 0.0001, one-sample *t*-test). The box represents the interquartile range with the median line. Whiskers extend from the 5th to 95th percentiles. Dots show individual data points beyond the whiskers. The dashed line at 100% indicates the reference value (normal side).

**Figure 3:**
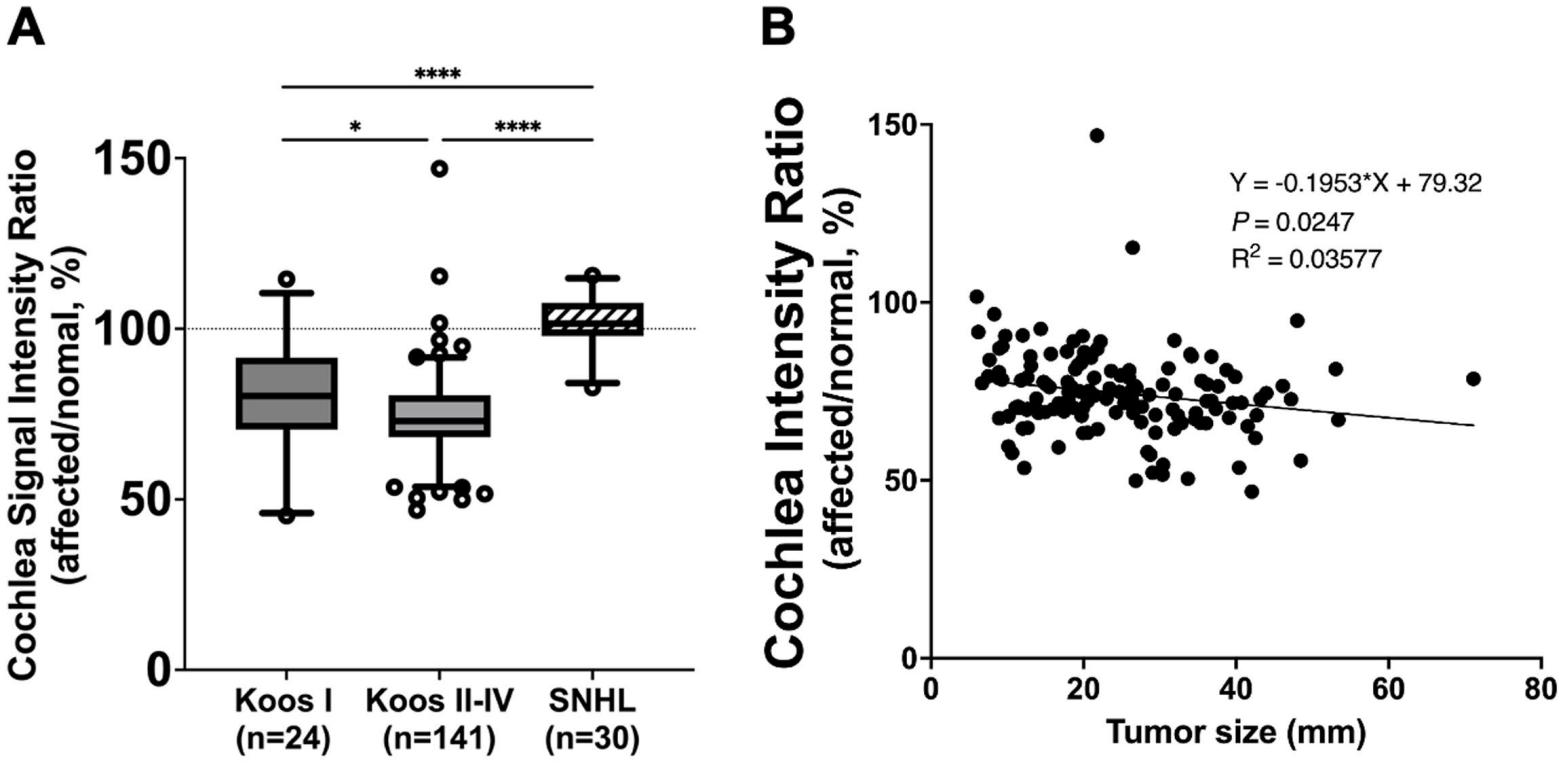
**(A)** Comparison of cochlea signal intensity ratio between vestibular schwannoma (VS) and unilateral sensorineural hearing loss (SNHL) groups. The VS group analyzed using pure tone averages (PTA) (Koos I: *n* = 24, Koos II–IV: *n* = 141) showed a significantly lower cochlea signal intensity ratio than the SNHL group (*n* = 30) (****p < 0.0001). The SNHL group did not show a significant difference from the reference value (100%). Comparison of cochlea signal intensity ratio between tumors classified as Koos I (*n* = 24) and those classified as Koos II–IV (*n* = 141). The Koos I group demonstrated significantly higher values compared to the Koos II–IV group. The box represents the interquartile range with the median line. Whiskers extend from the 5th to 95th percentiles. Dots show individual data points beyond the whiskers. Cochlea signal intensity ratio in relation to Koos classification and tumor size. **(B)** Linear regression analysis of tumor size and cochlea signal intensity ratio in the Koos II–IV group revealed a significant negative correlation, indicating that larger tumors were associated with lower cochlea signal intensity ratios. *p < 0.05.

In 161 out of 165 patients with VS, the mean signal intensity in the cochlea was lower on the affected side compared to the normal side. The affected side also showed a significantly lower cochlear signal intensity ratio (mean ± SD: 75.3% ± 13.0%) compared to the reference ratio (normal side), which had a constant value of 100% (*p* < 0.0001) (Figure 2). The group of patients with SNHL did not show a significant difference compared to the reference ratio. The VS group analyzed using PTA showed a significantly lower cochlear signal intensity ratio (mean ± SD: 75.3 ± 12.2%) compared to the SNHL group (mean ± SD: 101.7% ± 7.6%) (*p* < 0.0001) (Figure 3A). The correlation between tumor size and cochlear signal intensity ratio (affected/normal) was also evaluated. The Koos grade I group (*n* = 24) demonstrated significantly higher values (mean ± SD: 79.9% ± 15.9%) compared to the Koos grade II–IV group (*n* = 141) (mean ± SD: 74.5% ± 12.3%) (*p* < 0.05) (Figure 3A). Furthermore, a significant negative correlation was observed between tumor sizes and cochlea signal intensity ratios in the Koos grade II–IV group, indicating that larger tumors were associated with lower cochlea signal intensity ratios (Figure 3B).

**Figure 4:**
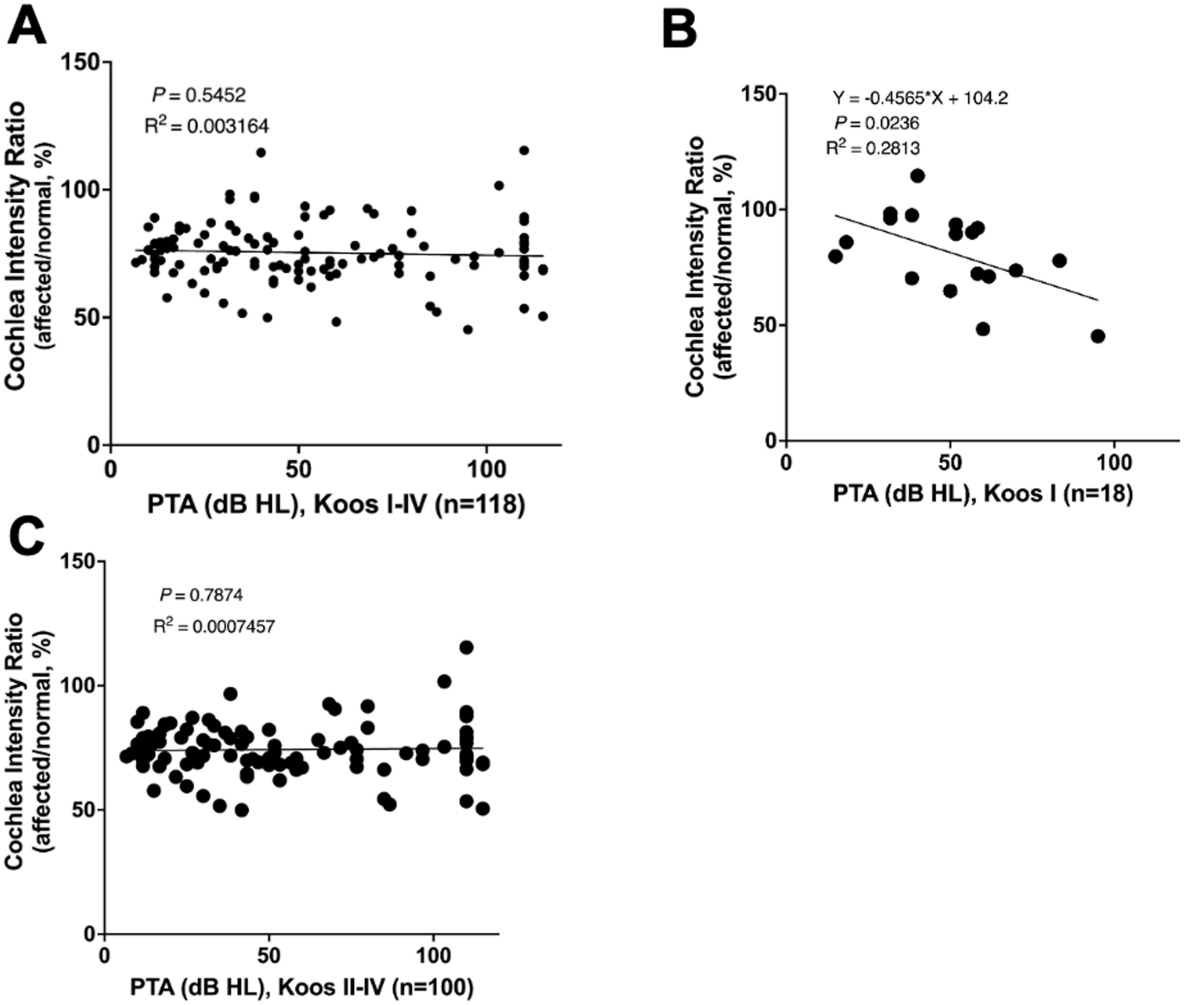
**(A)** Correlation analysis between cochlear signal intensity ratio and average hearing level in all patients with VS (*n* = 118). No significant correlation was found. **(B)** Correlation analysis between cochlear signal intensity ratio and average hearing level in Koos grade I tumors (*n* = 18). The cochlear signal intensity ratio was significantly lower in patients with poorer hearing levels. **(C)** Correlation analysis between cochlear signal intensity ratios and average hearing levels in Koos grade II–IV tumors (*n* = 100). No significant correlation was found.

A correlation analysis between cochlear signal intensity ratios and average hearing levels in patients with VS was also performed. Overall, no significant correlation was found among all VS patients (*n* = 118) (Figure 4A). However, in patients with Koos grade I tumors (*n* = 18), the cochlear signal intensity ratio was significantly lower in those with poorer hearing levels (Figure 4B), while in patients with Koos grade II–IV tumors (*n* = 100), no significant correlation was observed (Figure 4C).

**Figure 5:**
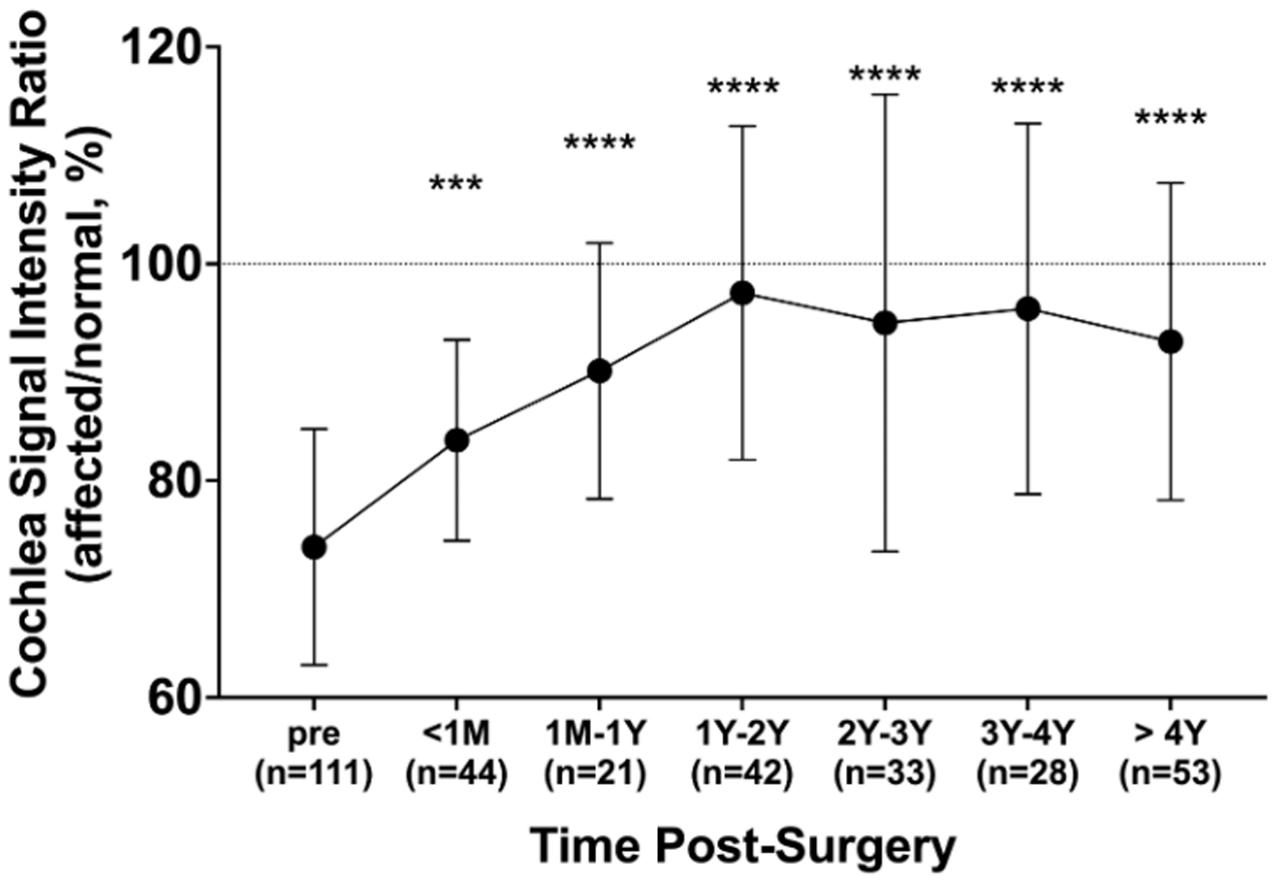
Changes in cochlea signal intensity ratio after surgery. The ratio was compared pre-surgery (*n* = 111) and at different post-surgery time points: within 1 month (n = 44), 1 month to 1 year (*n* = 21), 1–2 years (*n* = 42), 2–3 years (*n* = 33), 3–4 years (*n* = 28), and over 4 years (*n* = 53). All post-surgery groups exhibited significantly elevated ratios compared to before surgery. Error bars represent the standard deviation. ***p < 0.001, ****p < 0.0001.

The mean signal intensity ratio of the normal and affected cochlea (affected/normal) was evaluated in 111 patients whose tumors had been surgically removed. The mean signal ratio of the cochlea was evaluated for preoperative MRIs (*n* = 111), MRIs taken within 1 month postoperatively (*n* = 44), MRIs taken within 1 year postoperatively (*n* = 21), MRIs taken 1–2 years postoperatively (*n* = 42), MRIs taken 2–3 years postoperatively (*n* = 33), MRIs taken 3–4 years postoperatively (*n* = 28), and MRIs taken 4–10 years postoperatively (*n* = 53). Preoperatively, the mean signal intensity ratio of the cochlea was 73.9%; however, it recovered to 83.7% at 1 month postoperatively and to 97.3% at 1–2 years postoperatively (Figure 5). This improvement was statistically significant compared to the preoperative signal intensity ratio (*p* < 0.001).

Among the 42 patients who underwent MRI 1–2 years postoperatively, 26 patients had postoperative hearing tests. In that group, 4 out of 26 (15.4%) were classified as hearing preservation cases (all were Koos grade I VS) and 22 out of 26 (84.6%) were classified as hearing non-preservation cases. The signal intensity of the cochleae was compared between preoperative and postoperative measurements (post/preoperative). The ratio of change (mean ± SD) was 1.29 ± 0.09 in the hearing preservation group and 1.27 ± 0.203 in the hearing non-preservation group, with no significant difference between the two groups (*p* = 0.82).

## 4 Discussion

This study assessed the cochlear signal intensity in patients with VS using bFFE MRI sequences. Our findings demonstrated significant decreases in the signal intensity of the affected cochlea in patients with VS, but not in patients with SNHL. In the results of this study, the diagnostic accuracy of bFFE in patients with VS was 93.9% for sensitivity and 93.3% for specificity when the cut-off value was set at SIR 93%. This represents a higher sensitivity compared to previous reports using FLAIR (sensitivity: 80%, specificity: 95%) (7). Additionally, we identified a negative correlation between tumor size and cochlear signal intensity ratio in Koos grade II–IV tumors. Furthermore, we observed a normalization of the signal intensity difference following tumor resection. These results offer valuable insights into the impact of VS on cochlear physiology and have potential implications for the clinical management and monitoring of VS patients.

The bFFE sequences, also known as fast imaging employing steady-state acquisition (FIESTA) or true steady-state free precession (SSFP) (12) sequences, are characterized by their ability to rapidly capture images with suppressed flow artifacts. This technique enables visualization of subtle compositional changes in the cochlea without the use of contrast media, as the signal intensity is directly related to the T2/T1 ratio.

The decreased signal intensity observed in the cochleae affected by VS may indicate elevated protein concentrations. This finding is consistent with Silverstein et al.’s 1966 report, which documented protein levels 5–15 times higher than normal in VS cochleae (14). Recent studies suggest that various cytokines secreted by VS, such as tumor necrosis factor alpha and fibroblast growth factor 2, may affect the cochlea (3). Furthermore, the reduction in bFFE signal observed in patients with VS is specific to VS and is not observed in patients with CPA meningioma (15, 16), providing support for the hypothesis that these changes are unique to the substances secreted by VS.

Interestingly, only Koos grade I VS demonstrated a correlation between cochlear signal intensity ratios and hearing levels. This finding is consistent with the study by Kim et al. (10). The correlation may be attributed to the combined effects of tumor compression within the narrow internal auditory canal and secretions from VS. Moreover, the absence of this correlation in higher Koos grades suggests that the impact on hearing may plateau in larger tumors. In support of this, Anne et al. reported that hearing loss progresses along with the growth of Koos grade I VS; however, there is no difference in the rate of hearing loss between growing and non-growing tumors in Koos grades 2–4 (17).

In Koos grade II–IV VS, we observed a negative correlation between tumor size and cochlear signal intensity. This suggests that the cochlear signal intensity ratio may decrease as the tumor grows. This finding aligns with previous reports stating that cochlear protein concentration increased in large VS tumors that involved the brainstem compared to intracanalicular VS tumors (18). Additionally, we observed that the signal intensity of the affected cochlea recovered to the same level as the normal side about 1 year after tumor resection. This finding may be valuable for postoperative follow-up and early detection of VS recurrence. Given that the postoperative recurrence rates for VS vary widely from 5.5 to 44% (19–21) and recurrence has been reported even after total tumor resection (19), MRI follow-up is crucial. The normalization of signal intensity, regardless of hearing preservation, suggests that cochlear signal intensity may not directly reflect hearing level.

The cochlea and labyrinthine fibrosis are possible effects after surgery. The incidence rate varies depending on the report, but the highest incidence rate is reported to be with the trans-labyrinthine approach, while the incidence rate with the suboccipital approach is reported to be as low as 33% in cases of hearing loss (22–24). In this study, the signal intensity of the affected cochlea recovered to the same level as the normal side about 1 year after tumor resection. At our hospital, we perform all VS resections using the suboccipital approach. Therefore, although cochlear fibrosis may affect the signal, it is not the only factor influencing signal recovery; the normalization of the microenvironment, including protein levels within the cochlea, is also likely to play a role.

This study has several limitations. First, the small number of VS patients with Koos grade 1 tumors limits the generalizability of our findings to this subgroup. Second, accurately assessing the size of Koos grade I VS tumors proved to be challenging and posed a challenge to our analysis. These limitations may have affected our ability to fully elucidate the relationship between cochlear signal intensity, hearing function, and tumor characteristics in early-stage VS patients.

In conclusion, this study demonstrates that cochlear signal intensity using bFFE sequencing may reflect VS-affected changes in the cochlea. These findings have potential implications for tumor assessment, surgical planning, and postoperative monitoring in VS patients.

## Data Availability

The raw data supporting the conclusions of this article will be made available by the authors, without undue reservation.

## References

[1] Bridger MW Farkashidy J. The distribution of neuroglia and schwann cells in the 8th nerve of man. J Laryngol Otol. (1980) 94:1353–62. doi: 10.1017/S0022215100090186, PMID: 7441048

[2] Stangerup SE Caye-Thomasen P. Epidemiology and natural history of vestibular schwannomas. Otolaryngol Clin N Am. (2012) 45:257–68. doi: 10.1016/j.otc.2011.12.008, PMID: 22483814

[3] Dilwali S Landegger LD Soares VY Deschler DG Stankovic KM. Secreted factors from human vestibular schwannomas can cause cochlear damage. Sci Rep. (2015) 5:18599. doi: 10.1038/srep18599, PMID: 26690506 PMC4686978

[4] Roosli C Linthicum FH Jr Cureoglu S Merchant SN. Dysfunction of the cochlea contributing to hearing loss in acoustic neuromas: an underappreciated entity. Otol Neurotol. (2012) 33:473–80. doi: 10.1097/MAO.0b013e318248ee02, PMID: 22377650 PMC3302957

[5] Alyssa B Samuel E Sasa V Stankovic KM. Sporadic vestibular schwannoma size and location do not correlate with the severity of hearing loss at initial presentation. Front Oncol. (2022) 12:836504. doi: 10.3389/fonc.2022.836504, PMID: 35372070 PMC8965062

[6] Ren Y Chari DA Vasilijic S Welling DB Stankovic KM. New developments in Neurofibromatosis type 2 and vestibular schwannoma. Neurooncol Adv. (2020) 3:vdaa153. doi: 10.1093/noajnl/vdaa153, PMID: 33604573 PMC7881257

[7] Petrovic BD Futterer SF Hijaz T Russell EJ Karagianis AG. Frequency and diagnostic utility of intralabyrinthine FLAIR hyperintensity in the evaluation of internal auditory canal and inner ear pathology. Acad Radiol. (2010) 17:992–1000. doi: 10.1016/j.acra.2010.04.002, PMID: 20605731

[8] Miller ME Mafee MF Bykowski J Alexander TH Burchette RJ Mastrodimos B. Hearing preservation and vestibular schwannoma: intracochlear FLAIR signal relates to hearing level. Otol Neurotol. (2014) 35:348–52. doi: 10.1097/MAO.0000000000000191 24366469

[9] Lee IH Kim H-J Chung WH. Signal intensity of the labyrinth in patients with surgically confirmed or radiologically diagnosed vestibular schwannoma on isotropic 3D fluid-attenuated inversion recovery MR imaging at 3T. Eur Radiol. (2010) 20:949–57. doi: 10.1007/s00330-009-1626-9, PMID: 19898851

[10] Kim DY Lee JH Goh MJ Sung YS Choi YJ Yoon RG. Clinical significance of an increased cochlear 3D fluid-attenuated inversion recovery signal intensity on an MR imaging examination in patients with acoustic neuroma. AJNR Am J Neuroradiol. (2014) 35:1825–9. doi: 10.3174/ajnr.A3936, PMID: 24742808 PMC7966264

[11] Nathan CT Gabriela BD Polly H Kim J Diegnan B Go JL. Cochlear FLAIR signal changes in hearing preservation vestibular schwannoma surgery. Otol Neurotol. (2019) 40:375–83. doi: 10.1097/MAO.0000000000002102, PMID: 30664035

[12] Klaus S Stefan L. Principles and applications of balanced SSFP techniques. Eur Radiol. (2003) 13:2409–18. doi: 10.1007/s00330-003-1957-x, PMID: 12928954

[13] Koos WT Day JD Matula C Levy DI. Neurotopographic considerations in the microsurgical treatment of small acoustic neurinomas. J Neurosurg. (1998) 88:506–12. doi: 10.3171/jns.1998.88.3.0506, PMID: 9488305

[14] Silverstein H Schuknecht HF. Biochemical studies of inner ear fluid in man. Arch Otolaryngol. (1966) 84:395–402. doi: 10.1001/archotol.1966.00760030397003, PMID: 5921712

[15] Venkatasamy A Le Foll D Karol A Lhermitte B Charpiot A Debry C. Differentiation of vestibular schwannomas from meningiomas of the internal auditory canal using perilymphatic signal evaluation on T2-weighted gradient-echo fast imaging employing steady state acquisition at 3T. Eur Radiol Exp. (2017) 1:8. doi: 10.1186/s41747-017-0012-7, PMID: 29708179 PMC5909335

[16] Kazuhiro I Jun H Kouichirou O. Decreased vestibular signal intensity on 3D-FIESTA in vestibular schwannomas differentiating from meningiomas. Neuroradiology. (2013) 55:261–70. doi: 10.1007/s00234-012-1100-2, PMID: 23070536

[17] Anne L Gerard JJM Marjan HW Goedegebure A. Hearing loss progresses faster in patients with growing Intracanalicular vestibular schwannomas. Otol Neurotol. (2016) 37:1442–8. doi: 10.1097/MAO.0000000000001190, PMID: 27579837

[18] Yamazaki M Naganawa S Kawai H Nihashi T Fukatsu H Nakashima T. Increased signal intensity of the cochlea on pre- and post-contrast enhanced 3D-FLAIR in patients with vestibular schwannoma. Neuroradiology. (2009) 51:855–63. doi: 10.1007/s00234-009-0588-6, PMID: 19727694

[19] de Boer NP Böhringer S Koot RW Malessy MJA van der Mey AGL Jansen JC. A prediction model for recurrence after translabyrinthine surgery for vestibular schwannoma: toward personalized postoperative surveillance. Eur Arch Otorrinolaringol. (2022) 279:2905–13. doi: 10.1007/s00405-021-07244-z, PMID: 35020036 PMC9072472

[20] Abouzari M Goshtasbi K Sarna B Khosravi P Reutershan T Mostaghni N. Prediction of vestibular schwannoma recurrence using artificial neural network. Laryngoscope Investig Otolaryngol. (2020) 5:278–85. doi: 10.1002/lio2.362, PMID: 32337359 PMC7178452

[21] El-Kashlan HK Zeitoun H Arts HA Hoff JT Telian SA. Recurrence of acoustic neuroma after incomplete resection. Am J Otol. (2000) 21:389–92. doi: 10.1016/S0196-0709(00)80049-6, PMID: 10821553

[22] Feng Y Lane JI Lohse CM Carlson ML. Pattern of cochlear obliteration after vestibular schwannoma resection according to surgical approach. Laryngoscope. (2020) 130:474–81. doi: 10.1002/lary.27945, PMID: 30919457

[23] Hedjrat A Schwager K Hofmann E Behr R. Postoperative cochlear obliteration after retrosigmoid approach in patients with vestibular schwannoma. J Neurol Surg B Skull Base. (2018) 79:343–8. doi: 10.1055/s-0037-1608649, PMID: 30009114 PMC6043167

[24] Shapiro S Kemper N Jameson A Lipschitz N Hazenfield M Zuccarello M. Cochlear fibrosis after vestibular schwannoma resection via the middle cranial Fossa approach. Audiol Neurootol. (2022) 27:243–8. doi: 10.1159/000520782, PMID: 35378528

